# In Vitro Study of the Differential Anti-Inflammatory Activity of Dietary Phytochemicals upon Human Macrophage-like Cells as a Previous Step for Dietary Intervention

**DOI:** 10.3390/ijms251910728

**Published:** 2024-10-05

**Authors:** Antonio J. Ruiz-Alcaraz, Lorena Baquero, Paula Martínez Pérez-Munar, Alba Oliva-Bolarín, María A. Sánchez-Martínez, Bruno Ramos-Molina, María A. Núñez-Sánchez, Diego A. Moreno

**Affiliations:** 1 Department of Biochemistry, Molecular Biology B and Immunology, School of Medicine, University of Murcia, Regional Campus of International Excellence, 30120 Murcia, Spain; lorena.baqueroa@um.es (L.B.); paula.martinezp1@um.es (P.M.P.-M.); mariaantonia.martinez1@um.es (M.A.S.-M.); 2 Obesity and Metabolism Laboratory, Biomedical Research Institute of Murcia (IMIB), 30120 Murcia, Spain; alba.oliva@imib.es (A.O.-B.); bruno.ramos@imib.es (B.R.-M.); 3 Grupo Laboratorio de Fitoquímica y Alimentos Saludables (LabFAS), CEBAS-CSIC, Campus Universitario de Espinardo-25, 30100 Murcia, Spain; dmoreno@cebas.csic.es

**Keywords:** bioactive phytochemicals, anti-inflammatory agents, inflammatory diseases, glucosinolates, isothiocyanate, anthocyanins, phenolic acids

## Abstract

Chronic inflammatory diseases pose a substantial health challenge globally, significantly contributing to morbidity and mortality. Addressing this issue requires the use of effective anti-inflammatory strategies with fewer side effects than those provoked by currently used drugs. In this study, a range of phytochemicals (phenolic di-caffeoylquinic acid (Di-CQA), flavonoid cyanidin-3,5-diglucoside (Cy3,5DiG), aromatic isothiocyanate sinalbin (SNB) and aliphatic isothiocyanate sulforaphane (SFN)) sourced from vegetables and fruits underwent assessment for their potential anti-inflammatory activity. An in vitro model of human macrophage-like cells treated with a low dose of LPS to obtain a low degree of inflammation that emulates a chronic inflammation scenario revealed promising results. Cell viability and production of the key pro-inflammatory cytokines were assessed in the presence of various phytochemicals. The compounds Di-CQA and Cy-3,5-DiG, within low physiologically relevant doses, demonstrated notable anti-inflammatory effects by significantly reducing the production of key pro-inflammatory cytokines TNF-α and IL-6 without affecting cell viability. These findings underscore the potential of plant-derived bioactive compounds as valuable contributors to the prevention or treatment of chronic inflammatory diseases. These results suggest that these compounds, whether used individually or as part of natural mixtures, hold promise for their inclusion in nutritional interventions designed to mitigate inflammation in associated pathologies.

## 1. Introduction

Human pathologies related to chronic inflammation represent relevant social and medical burdens, causing high morbidity and mortality worldwide [1]. The inflammatory process is part of the natural response of the organism to maintain homeostasis against external insults and tissue damage, playing a major role in tissue repair. However, if this acute process does not resolve properly, it may evolve into a chronic inflammatory scenario, which in turn may cause the appearance of different related diseases, such as rheumatoid arthritis and diabetes mellitus, among others [2]. In this aggravation of the pathophysiological situation, a series of cells, such as macrophages, and soluble mediators produced by immune system cells, including pro-inflammatory cytokines tumor necrosis factor alpha (TNF-α) and interleukin 6 (IL-6), play a key role [3,4].

Currently, non-steroidal anti-inflammatory drugs, or NSAIDs, represent the preferred treatment for inflammatory diseases, since they are able to inhibit the production of important proinflammatory mediators such as prostaglandins [5]. However, there is a great effort to develop alternatives to NSAIDs, as many of these agents present a variety of adverse effects, including cardiovascular, gastrointestinal, and renal toxicity [6,7]. This fact encourages the search for new natural therapeutic approaches with fewer side effects that may be of use as coadjuvants in the treatment of inflammatory diseases. In this regard, bioactive phytochemicals and ingredients derived from them, obtained from fruits and vegetables from the diet, have great anti-inflammatory potential and may contribute to preventing or even treating these pathologies [8].

Glucosinolates (GSLs) are amino acid-derived compounds classified according to their precursor amino acid (aliphatic-Met, Ala, Leu, Ile, or Val; aromatic–Phe or Tyr; and indolics, from Trp). Aliphatic GSLs are predominant in broccoli and cabbages (glucoraphanin, the parental GSL of sulforaphane (SFN)). On the other hand, mustards, especially oil-rich varieties, are rich in aromatic GSL, mainly glucosinalbin, and its hydrolysis yields, 4-hydroxybenzyl isothiocyanate, the compound responsible for its sharp flavor [9,10]. Another relevant mustard GSL is sinalbin (SNB), which, to the best of our knowledge, has not been mentioned in any publication related to human inflammation to the present date. The evaluation of the potential bioactivity of SNB as an anti-inflammatory agent would be useful in discriminating the specific bioactivity of isothiocyanates and GSL.

On the other hand, one of the most important representatives of bioactive isothiocyanates in human health is SFN, which has been described to exert its main function through the nuclear factor erythroid 2–related factor 2 (NRF2) intracellular pathway. SFN binds to KEAP1 (Kelch-like ECH-associated protein 1), the natural suppressor of NRF2, allowing the transcription of Phase II metabolism enzymes [11]. Furthermore, SFN is also able to interfere with the activity of the nuclear factor kappa-light-chain-enhancer of activated B cells (NFκB), decreasing its capability to bind target sequences of genes related to inflammatory processes, such as those codifying for TNF-α [12]. Thus, this ingredient can be taken as a gold standard of a bioactive anti-inflammatory natural compound to which compare the potency of novel potentially interesting phytochemicals.

Chlorogenic acids are very ubiquitous in nature and in plant-origin foods (vegetables and fruits, e.g., artichokes, berries), classically studied for their antioxidant capacity [13,14], and, more recently, evaluated for their potential anti-inflammatory effects mediated by the inhibition of related key enzymes (e.g., COX-2) and the release of pro-inflammatory cytokines (e.g., TNF-α, IL-6, and IL-1β) [15,16]. Similarly, colored flavonoids (anthocyanins), including acylated cyanidins such as those found in Brassicas and berries (e.g., cyanidin-3,5-diglucoside, Cy3,5DiG) have also been found to exert antioxidant, anti-inflammatory, anticancer, anti-diabetic, and cardiovascular protective activity [17]. Di-glycosylated anthocyanins diminished the induction of pro-inflammatory cytokines such as IL-1β, TNF-α, and IL-6, which awakened the interest in evaluating structure–activity relationships between these substances and their potential as agents for preventing inflammation and related conditions [16].

The hypothesis of this study is that several phytochemicals present in vegetables and fruits (e.g., green leafy vegetables, tropical fruits, etc.) may present relevant bioactivity of interest for human health as anti-inflammatory agents, and to prove this hypothesis, we tested and verified the capacity of single compounds to inhibit the production of key pro-inflammatory cytokines TNF-α and IL-6 by human macrophage-like cells in vitro within low physiologically-relevant dosing and without any cytotoxic effects. This preliminary evaluation supposes a basic requirement for additional research on the potential synergistic effects of combinations of the bioactive compounds for the design of further investigations at the preclinical and clinical levels.

## 2. Results

### 2.1. Effect of Phytochemicals Sulforaphane, Sinalbin, Di-Caffeoylquinic Acid and Cyanidin-3,5-Diglucoside on Human Macrophage-like Cell Viability

None of the tested compounds showed a significant effect on the viability of the human macrophage-like HL-60 differentiated cells in this in vitro model of low-degree inflammation at any of the tested doses (10 µM, 25 µM, and 50 µM). The only exception was SFN, which produced a significant reduction of about 30.4% in cell viability , but only at its highest dose (50 µM). This result was not unexpected as it was similar to what was observed in our previous studies [17] (Table 1).

### 2.2. Effect of Phytochemicals Sulforaphane, Sinalbin, Di-Caffeoylquinic Acid, and Cyanidin-3,5-Diglucoside on TNF-α Production

**Figure 1:**
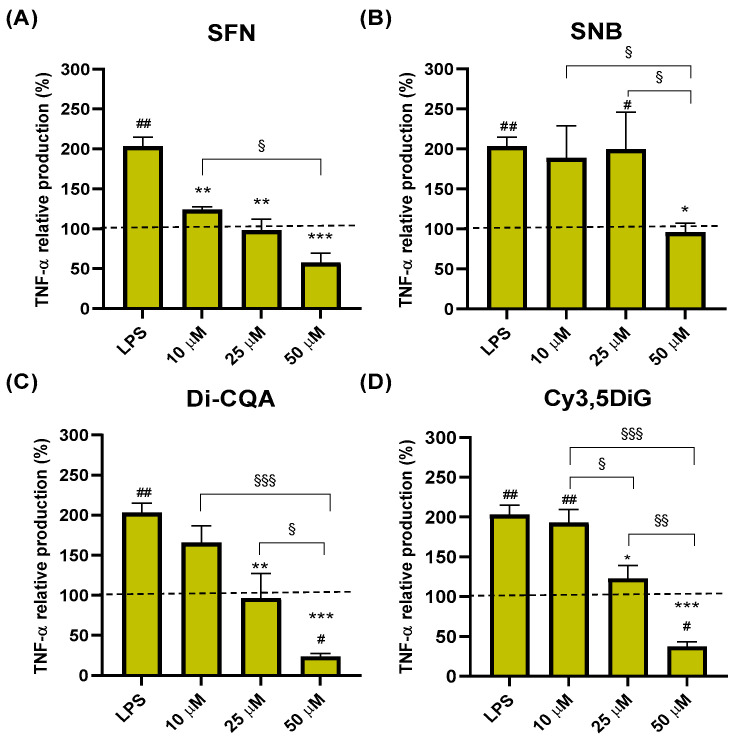
Effect of Sulforaphane, Sinalbin, Di-caffeoylquinic acid, and Cyanidin-3,5-diglucoside on TNF-α production. Data are presented as percentage compared to the negative control (no treatment) valued as 100% from three in vitro assays with different doses (10 µM, 25 µM and 50 µM) of SFN (**A**), SNB (**B**), Di-CQA (**C**) and Cy3,5DiG (**D**) for 24 h in the presence of LPS (0.1 µg/mL) as a pro-inflammatory stimulus (N = 3). Values of *p* were calculated using the ANOVA test with a post hoc Tukey’s HSD test. Statistically significant differences are represented as: * *p* < 0.05, ** *p* < 0.01 and *** *p* < 0.001, compared to LPS treatment; # *p* < 0.05 and ## *p* < 0.01, compared to negative control; ^§^ *p* < 0.05, ^§§^ *p* < 0.01 and ^§§§^ *p* < 0.001, for comparisons between different doses of compounds. Dot lines represent cytokine normalized level of 100% corresponding to negative control. SFN, Sulforaphane; SNB, Sinalbin; Cy3,5DiG, Cyanidin-3,5-diglucoside; Di-CQA, Di-caffeoylquinic acid; LPS, lipopolysaccharide; TNF-α, tumor necrosis factor alpha.

To evaluate the anti-inflammatory potential of the phytochemicals selected, we performed an ELISA analysis to determine the effect of the different compounds on the production of TNF-α and IL-6 in an LPS-induced inflammatory model of HL-60 cells. Our results showed that after 24 h, the treatment of macrophage-like differentiated HL-60 cells with SFN significantly reduced the LPS-induced production of TNF-α in a dose-dependent manner (*), lowering TNF-α production to similar levels as those observed in cells not exposed to LPS (negative control, cytokine normalized level of 100% represented as a dotted line) (Figure 1A). Furthermore, significant differences were also observed in TNF-α levels between the lowest (10 µM) and the highest (50 µM) doses (^§^ *p* = 0.03) (Figure 1A). On the other hand, the exposure of the cells to the aliphatic GSL SNB significantly reduced the production of TNF-α induced by LPS only with the highest tested dose of 50 µM down to basal levels (* *p* = 0.02) (Figure 1B). Regarding the hydroxycinnamic acid Di-CQA, the results showed a significant reduction in the LPS-induced TNF-α production both at 25 μM and 50 μM doses after 24 h treatment (down to 97%, ** *p* < 0.01; and 24%, *** *p* < 0.001; respectively) following a dose-response trend with significant differences between the higher dose (50 µM) and the lower doses tested (^§^ *p* < 0.05 compared to 25 µM, ^§§§^ *p* < 0.001 compared to 10 μM) (Figure 1C). Remarkably, the reduction in TNF-α levels in cells treated with 50 µM Di-CQA was also significant compared to the negative control of untreated cells (^#^ *p* < 0.05) (Figure 1C). Similarly, the cells exposed to the anthocyanin Cy3,5DiG showed a similar pattern as those treated with Di-CQA, with significant reductions in TNF-α levels after 24 h of treatments with 25 and 50 µM, showing an increasing dose-response effect with significant differences between all the tested doses (^§^ *p* < 0.05 for 10 µM vs. 25 µM; ^§§^ *p* < 0.01 for 25 µM vs. 50 µM; and, ^§§§^ *p* < 0.001 for 10 µM vs. 50 µM), and a further significant reduction with the highest dose of 50 µM compared to control non-inflammatory conditions (^#^ *p* < 0.05) (Figure 1D).

### 2.3. Effect of Phytochemicals Sulforaphane, Sinalbin, Di-Caffeoylquinic Acid, and Cyanidin-3,5-Diglucoside on IL-6 Production

**Figure 2:**
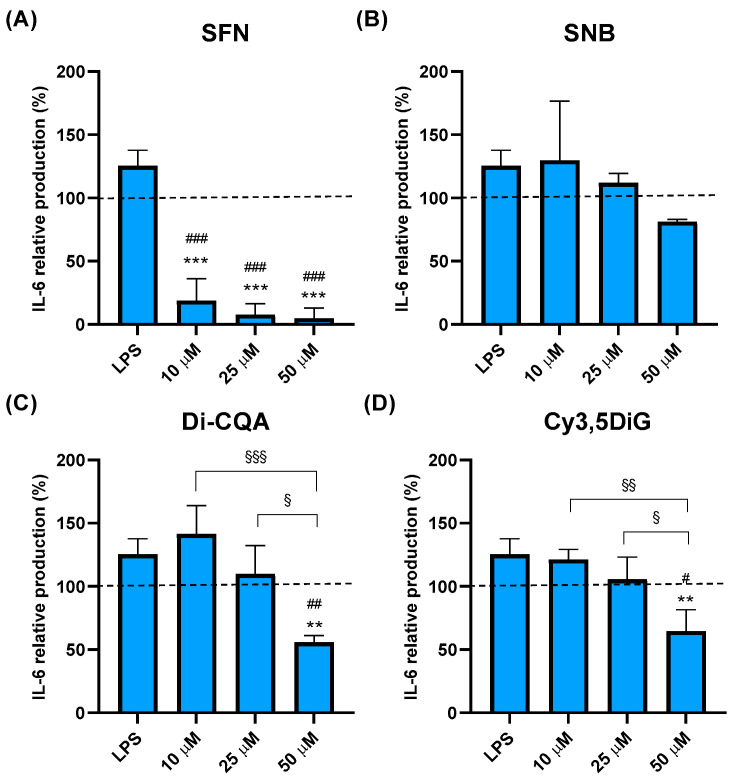
Effect of Sulforaphane, Sinalbin, Di-caffeoylquinic acid, and Cyanidin-3,5-diglucoside on IL-6 production. Data are presented as percentages compared to the negative control (no treatment) valued as 100% from three in vitro assays with different doses (10 µM, 25 µM, and 50 µM) of SFN (**A**), SNB (**B**), Di-CQA (**C**), and Cy3,5DiG (**D**) for 24 h in the presence of LPS (0.1 µg/mL) as a pro-inflammatory stimulus (N = 3). Values of *p* were calculated using the ANOVA test with a post hoc Tukey’s HSD test. Statistically significant differences are represented as ** *p* < 0.01 and *** *p* < 0.001 compared to LPS treatment; # *p* < 0.05, ## *p* < 0.01 and ### *p* < 0.001 compared to the negative control; and ^§^ *p* < 0.05, ^§§^ *p* < 0.01 and ^§§§^ *p* < 0.001 for comparisons between different doses of compounds. Dotted lines represent normalized cytokine levels of 100% corresponding to the negative control. SFN, sulforaphane; SNB, sinalbin; Cy3,5DiG, cyanidin-3,5-diglucoside; Di-CQA, di-caffeoylquinic acid; IL-6, interleukin 6; LPS, lipopolysaccharide.

The exposure of macrophage-like cells to SFN significantly and drastically reduced the LPS-induced levels of IL-6 at all the tested concentrations (Figure 2A). However, contrary to the dose-dependent effect observed in SFN-treated cells in TNF-α production, even the exposure to the lower dose of 10 μM was already sufficient to reach the maximum reduction in IL-6 production after 24 h (Figure 2A). Furthermore, such reduction was not only significantly lower than that observed in LPS-stimulated proinflammatory *conditions* (*** *p* < 0.001) but also compared to basal conditions of non-stimulated cells (^###^ *p* < 0.001, dotted line) (Figure 2A). On the other hand, the treatment of cells with SNB did not significantly affect the LPS-induced production of IL-6 at any of the tested doses (Figure 2B). In the case of Di-CQA, the production of IL-6 was only affected by the higher dose of 50 μM, lowering this cytokine levels by about 50% when compared to the negative control of unstimulated cells (^##^ *p* < 0.01) and 75% compared to LPS-stimulated cells (** *p* < 0.01) (Figure 2C). On the other hand, no effect was observed after exposure to the 10 μM and 25 μM doses, and, furthermore, the effect of the 50 µM dose was also significantly lower compared to the 10 µM and 25 µM doses (^§§§^ *p* < 0.001 for 10 µM vs. 50 µM; and, ^§^ *p* < 0.05 for 25 µM vs. 50 µM) (Figure 2C). Finally, the treatment of cells with the anthocyanin Cy3,5DiG showed a similar pattern to that of Di-CQA since only the treatment with the dose of 50 µM significantly reduced the production of IL-6 compared to both LPS-stimulated (** *p* < 0.01) and basal conditions (^#^ *p* < 0.05), while no effect was observed with minor doses of Cy3,5DiG. Furthermore, such an effect of the 50 µM dose of Cy3,5-CQA was also significantly lower when compared to the treatments with 10 µM and 25 µM (^§§^ *p* < 0.01 for 10 µM vs. 50 µM; and ^§^ *p* < 0.05 for 25 µM vs. 50 µM) (Figure 2D).

## 3. Discussion

Herein, we have assessed, for the first time, the potential anti-inflammatory bioactivity of a series of phytochemicals in a well-established in vitro model of human macrophage-like cells obtained from the differentiation of HL-60 cells maintained in a state of a low degree of inflammation after stimulation with a low dose of LPS for 24 hours that emulates chronic inflammation scenario. Our results show that two of those compounds, Di-CQA and Cy-3,5-DiG, promote remarkable anti-inflammatory activity, since both were capable of reducing the production of the systemic pro-inflammatory cytokines TNF-α and IL-6. These results support the use of plant-derived bioactive compounds (e.g., nutraceuticals, functional ingredients) to help prevent or treat chronic human inflammatory diseases [8]. Furthermore, this might help design natural mixtures of bioactive phytochemicals or determine single bioactive compounds that could be used as a new phytotherapeutic approach to treat inflammation-related pathologies.

The in vitro model used in this study, which was previously established and optimized by us, provides a reliable method to study the anti-inflammatory potential of compounds from vegetal origin in a scenario of low-degree inflammation resembling that found in several chronic inflammatory diseases [18]. Thus, we used this model to study the anti-inflammatory bioactivity of potentially active compounds present not only in cruciferous foods (e.g., sprouts, baby leaf varieties, cabbages, etc.) but also in a myriad of fruits and vegetables as sources of dichlorogenic acids (e.g., berries, artichoke, coffee, apples, etc.) in order to assess their potential to reduce the production and release of the systemic key pro-inflammatory cytokines TNF-α and IL-6. In addition, we included in this study a treatment with SFN as a positive control, as it is considered a gold standard of anti-inflammatory bioactive compounds from plant origin [19,20], and whose inflammatory activity has been confirmed here.

Our results showed that the new compounds tested had no effect on cell viability at any dose, thus showing no cytotoxicity whatsoever, while exerting high anti-inflammatory activity at the higher concentration tested, which was reflected in their ability to significantly reduce the production of TNF-α and IL-6, reducing the levels of these pro-inflammatory cytokines below the levels observed in control conditions of untreated cells. Furthermore, the hydroxycinnamic acid Di-CQA and the anthocyanin Cy3,5DiG were able to block the production of both TNF-α and IL-6 at basal levels when cells were treated with lower doses. It was also noteworthy that, at its highest dose (50 µM), Di-CQA was able to further reduce the production of TNF-α to a lower level than that observed with the gold standard SFN. Overall, the capacity of these compounds to efficiently inhibit the production of TNF-α and IL-6, especially Di-CQA and Cy3,5DiG, highlights their potential as anti-inflammatory agents capable of reducing the production of these pro-inflammatory factors by human macrophages that play a major function as inductors and maintainers of chronic inflammation in many related pathologies [21].

To the best of our knowledge, this is the first study reporting the anti-inflammatory effect of Di-CQA as a single agent capable of reducing the production and release of the key pro-inflammatory cytokines TNF-α and IL-6 by human macrophages in an in vitro model of differentiated HL-60 cells. Our data are in agreement with those reported by others who have shown the potential of different plant foods and derived products rich in chlorogenic acid derivatives, including Di-CQA, as anti-inflammatory agents and analgesics used in traditional medicine [22,23]. Furthermore, the results are also in line with previous data showing that complex leaf extracts rich in Di-CQA obtained from *Artemisia selengensis Turcz* could alleviate gout inflammation in an in vitro model of THP-1 cells by inhibiting the NLRP3 inflammasome and activating the NRF2 signaling pathway and thus the production of several pro-inflammatory cytokines [24]. This anti-inflammatory effect may be produced not only by the Di-CQA present in the leaf extracts but also by the many other phytochemicals present in the mix. Thus, our results now confirm that Di-CQA has a potent anti-inflammatory activity, per se, when used as a single agent.

Anthocyanins have been widely studied for their relevant bioactivity. The available literature supports the multiple functions of different natural products containing these types of compounds [25,26], with particular interest in the already proven bioactivity of their major representative, cyanidin-3-O-glucoside (C3G) [27]. This compound has not only been described as an anti-inflammatory agent [18,28], but it can also act as a potential anti-diabetic [29,30], anti-cancer [31], and antioxidant compound, among other functions [32,33,34]. In this study, we have focused on the analysis of a less-known anthocyanin, Cy3,5DiG, which has been previously predicted in silico to be one of the bioactive agents responsible for the reduction in the levels of IL-6, and, therefore, of the anti-inflammatory activity, of *Ficus carica* [35]. Here, we have confirmed the capacity of Cy3,5DiG to reduce not only the production of IL-6 by human macrophages in vitro as a single agent but also the production of TNF-α, one of the other major pro-inflammatory cytokines mediating inflammatory-related pathologies such as Crohn’s disease (CD) [36] and rheumatoid arthritis (RA) [37].

Finally, the aromatic isothiocyanate SNB, which has been shown before to participate in the anti-proliferative and antimicrobial effects of mustard (*Sinapis*) extracts [38], showed the lowest anti-inflammatory bioactivity in our in vitro model of human macrophages. Although it was able to block the production of TNF-α and IL-6 when cells were treated with the highest dose tested, the level of inhibition reached by SNB was the lowest amongst the tested compounds. Nevertheless, this is the first time that SNB has been proven to present anti-inflammatory bioactivity upon human macrophages as a single agent, which adds to its capacity of acting as an antioxidant agent, which could make SNB an interesting phytochemical to consider for nutritional interventions [39].

Altogether, our results show how the studied phytochemicals may function individually as single agents with remarkable anti-inflammatory activities at low doses (≤50 µM), which would be like those expected to be found in physiological conditions after the intake of food matrices rich in these bioactive ingredients. Thus, our data would be relevant to optimizing the incorporation of interesting “phytochemical cocktails” from vegetables and fruits, natural sources of the studied compounds, to nutritional interventions addressing the reduction in inflammation in related chronic pathologies.

## 4. Materials and Methods

### 4.1. Compounds

Phenolic di-caffeoylquinic acid (Di-CQA), flavonoid Cy3,5DiG, and aromatic isothiocyanate SNB were obtained from Phytoplan Diehm & Neuberger GmbH (Heidelberg, Germany), while the aliphatic isothiocyanate SFN was obtained from LKT Laboratories, Inc. (St. Paul, MN, USA) (Table 2).

### 4.2. Compound Preparation for In Vitro Assays

A 1 mg/mL stock solution of the different compounds was freshly prepared directly in sterile phosphate-buffered saline (PBS) pH = 7.4 (Biowest, Nuaillé, France), with the only exception of SFN, which was first diluted in dimethyl sulfoxide (DMSO) (Merck, Whitehouse Station, NJ, USA), in order to get a final stock solution of 1 mg/mL SFN in PBS containing 10% DMSO. The subsequent dilutions were made at the appropriate concentration in a complete cell-culture medium (CCM) prior to being used in the in vitro assays.

### 4.3. Cell Culture

Phytochemical bioactivity was tested using macrophage cells obtained from the differentiation of the human acute myeloid leukemia cell line HL-60 (ATCC^®^ CCL-240™, American Type Culture Collection, Rockville, MD, USA) as previously described [40]. In brief, undifferentiated HL-60 cells were first kept growing at an exponential ratio in CCM, RPMI-1640 (Biowest, Nuaillé, France), supplemented with 10% fetal bovine serum (GIBCO Invitrogen, Paisley, UK) and 1% penicillin/streptomycin (GIBCO) prior to their differentiation. Growing conditions were set at 37 °C and under an atmosphere of 5% CO_2_ in the incubator. All the in vitro assays were performed on cells always differentiated between passages 5 and 20.

### 4.4. Cell Viability Assays

The viability of human macrophage-like cells obtained by the differentiation of HL-60 and treated with different doses of the tested phytochemicals was measured by MTT assay [37]. Briefly, after cell differentiation with 10 ng/mL phorbol myristate acetate (PMA; Sigma Chemical Co., St. Louis, MO, USA) in 96-well plates, the cells were pre-incubated with 10, 50, and 100 μg/mL of either of the tested phytochemicals, and after 30 min, the cells were treated with LPS (0.1 μg/mL) and incubated for 24 h. MTT (Alfa Aesar, Thermo Fisher, Karlsruhe, Germany) was then added to the final concentration of 483 μM and incubated for 1 h at 37 °C and under a 5% CO_2_ atmosphere. Then, an acidified isopropanol solubilization solution of 0.04 M hydrochloric acid and 0.1% NP-40 detergent was added to each well to lysate the cells and dissolve the purple formazan crystal formed inside the cells, obtaining a purple-colored solution. The absorbance was measured at 550 nm in a plate-reading spectrophotometer (Spectrostar Nano; BMG Labtech, Ortenberg, Germany). The cell viability was determined by comparison with control conditions, setting the latter as 100% viability and thus 0% cytotoxicity. Control cells were maintained under basal conditions and treated only with CCM. Three replicates were performed for each experimental condition.

### 4.5. In Vitro Anti-Inflammatory Assays

The anti-inflammatory bioactivity of the phytochemicals was analyzed as previously described [17]. Briefly, the potential to modulate the secretion of pro-inflammatory cytokines in inflammatory-like conditions induced by bacterial lipopolysaccharide (LPS) in vitro was studied in a model of human macrophage-like cells obtained by the differentiation of HL-60 cells (human myeloid leukemia) with a treatment of 10 ng/mL of PMA (Sigma Chemical Co., St. Louis, MO, USA) for 24 h upon a ratio of 2·10^5^ cells/well in 96-wells plates in CCM. Cells rested then in CCM without PMA for another 24 h. After differentiation and resting periods, tested compounds (or vehicles) were added to cells at doses of 10 μM, 25 μM, and 50 μM, and plates were pre-incubated for 30 min at 37 °C. Finally, LPS (Escherichia coli O111:B44; Sigma Chemical Co.) was added at a final concentration of 0.1 µg/mL to induce a low degree of pro-inflammatory conditions like those found in chronic inflammatory diseases. Plates were then incubated at 37 °C with 5% CO_2_ for 24 h, and supernatants were collected and kept at −20 °C until further analysis. Three replicates were performed for each experiment.

### 4.6. Enzyme-Linked Immunosorbent Assays

Stored supernatants from in vitro assays performed on human macrophage-like HL-60 cells were used to determine the levels of the pro-inflammatory cytokines TNF-α and IL-6 using colorimetric enzyme-linked immunosorbent assay (ELISA) kits (Invitrogen, Thermo Fisher Scientific, Waltham, MA, USA). The absorbance in each well was determined using a microplate reader (Spectrostar Nano; BMG Labtech, Ortenberg, Germany) at 450 nm and corrected at 570 nm. Each cytokine concentration was calculated using the corresponding standard curve. The modulation of cytokines was attained by calculating the level of release inhibition when compared to control conditions. To compare the effect of the different tested phytochemicals, the concentrations of the cytokines obtained from enzyme-linked immunosorbent assays (ELISA) (within the ranges 40–560 pg/mL for TNF-α and 1.5–110 pg/mL for IL-6) were normalized and represented as the percentage of cytokine production for each individual assay by setting the negative control of the corresponding experiment as the reference, valued as 100%. The effect of the tested compounds upon cytokine levels in inflammatory-like conditions induced by LPS is presented as the percentage fold-change of normalized results compared to the negative control (100%, represented as a dotted line in the result figures).

### 4.7. Statistical Analysis

Data are reported as normalized percentages compared to the negative control (100%). Results are reported as the mean ± SEM and are graphically represented as histograms. Calculations were performed using GraphPad Prism 9 software. Statistical differences were analyzed by ANOVA and Tukey’s HSD tests. Values of *p* under 0.05 were considered to indicate statistical significance.

## 5. Conclusions

In conclusion, some of the food phytochemicals individually tested in this study (SFN, Di-CQA, and Cy3,5DiG) have shown to be potentially active anti-inflammatory agents from a natural origin, as they were able to inhibit the production of key pro-inflammatory cytokines responsible for the establishment and maintenance of systemic chronic inflammation. Therefore, these data would be of great interest for the design of nutritional interventions that aim to ameliorate inflammatory diseases.

## Figures and Tables

**Table 1:** Effect of phytochemicals Sulforaphane, Sinalbin, Di-caffeoylquinic acid and Cyanidin-3,5-diglucoside on human macrophage-like HL-60 cell viability under low degree inflammatory conditions.

		Dose (µM)	
Compound	10	25	50
SFN	117.0 ± 13.1	101.8 ± 11.4	69.6 ± 9.51 *
SNB	123.9 ± 13.5	122.9 ± 8.8	126.6 ± 10.2
Di-CQA	138.5 ± 4.4	130.9 ± 8.4	120.2 ± 8.7
Cy3,5DiG	117.9 ± 5.7	106.5 ± 7.1	95.5 ± 6.8

Results represent viability (%) as the mean ± SEM following 24 h exposure of HL-60 differentiated cells to the tested compounds at the different doses indicated plus LPS (0.1 µg/mL). Data were obtained from three different assays (N = 3). * *p* < 0.05. Cy3,5DiG: cyanidin-3,5-diglucoside; Di-CQA: di-caffeoylquinic acid; SFN: sulforaphane; SNB: sinalbin.

**Table 2:** Compounds tested as anti-inflammatory agents.

Compound	Type	Abbreviation	Formula	Structure	Natural/Food Source
Sulforaphane	Aliphatic Isothiocyanate	SFN	C_6_H_11_NOS_2_	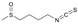	Glucoraphanin from broccoli, broccolini, cabbage, etc.
Sinalbin	Aromatic Isothiocyanate	SNB	C_14_H_18_NO_10_S_2_K	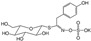	Glucosinalbin from mustards, horseradish, watercress, etc.
Di-caffeoylquinic acid	Hydroxycinnamic acid (phenolic acid)	Di-CQA	C_25_H_24_O_12_	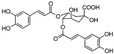	Chlorogenic acids in mustards, artichoke, eggplant, bay leaf, etc.
Cyanidin-3,5-diglucoside	Anthocyanin (flavonoid)	Cy3,5DiG	C_27_H_31_O_16_Cl	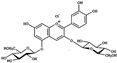	Natural pigments in berries, beans, sprouts, etc.

## Data Availability

All research data are presented in the article.
